# Supplementary material for: Physicochemical Characteristics, Techno-Functionalities, and Amino Acid Profile of Prionoplus reticularis (Huhu) Larvae and Pupae Protein Extracts
Source: Foods. 2023 Jan 16;12(2):417. doi: 10.3390/foods12020417 (PMC9857897; doi:10.3390/foods12020417)
Supplement: Supplementary file 1 [file foods-12-00417-s001.zip › foods-2069285-supplementary.pdf]

Supplementary Materials:

Table S1: Yield and recovery (%, dry weight basis) of HLPE and HPPE.

	HLPE	HPPE
Yield (%, dry weight)	31.9[b] ± 0.2	33.5[a] ± 0.3
Recovery (%, dry weight)	72.1[a] ± 4.0	76.5[b] ± 1.5

All values are expressed as mean ± standard deviation (SD); n=3; Means with different superscripts ([a-b]) within each row are significantly different ($p<0.05$), as determined by the posthoc Tukey's Honestly Significant Difference (HSD) test.

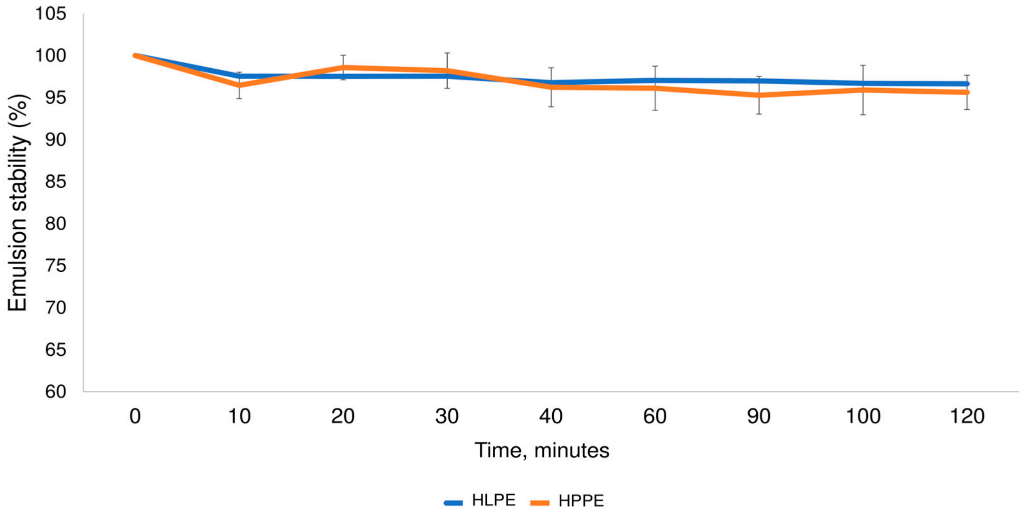

Figure S1. Emulsion stability of HLPE and HPPE.
